# Clinicopathological and genetic features of Zinner’s syndrome: two case reports and review of the literature

**DOI:** 10.3389/fruro.2023.1257368

**Published:** 2023-10-19

**Authors:** Ruijie Dai, Fan Jiang, Junjie Fan, Dalin He, Lei Li, Kaijie Wu

**Affiliations:** ^1^ Department of Urology, The First Affiliated Hospital of Xi’an Jiaotong University, Xi’an, Shaanxi, China; ^2^ Department of Urology, Baoji Central Hospital, Baoji, Shaanxi, China

**Keywords:** Zinner’s syndrome, seminal vesicle cyst, unilateral renal dysplasia, genetic mutation, exon sequencing

## Abstract

Zinner’s syndrome (ZS) is a rare congenital malformation due to abnormal development of the urogenital tract. It is characterized by the triad of unilateral renal agenesis, ipsilateral seminal vesicle cyst and ipsilateral ejaculatory duct obstruction. Cases are rarely reported in China since the incidence of the disease is low. Symptoms also vary widely among patients and its etiology is unclear. In this article, we described two patients with totally different cinicopathological and genetic features based on exon sequencing.

## Introduction

Zinner’s syndrome is a triad of mesonephric duct anomalies comprising unilateral renal agenesis, seminal vesicle cyst, and ejaculatory duct obstruction. It is a rare urogenital congenital developmental anomaly that occurs in early embryogenesis and affects the distal portion of the Wolffian duct (1). The syndrome usually presents mainly in the second and third decade of life (after the beginning of sexual activity) and is accompanied by prostatitis, painful ejaculation, hematospermia, perineal pain or discomfort, and sometimes infertility (2, 3). Due to the non-specificity of clinical symptoms, there are difficulties in the diagnosis of the disease, which also cause obstacles to the selection of correct treatment measures for Zinner’s syndrome. Therefore, accumulating experience in diagnosis and treatment of the disease is beneficial to the patient group. This paper introduces two patients with Zinner’s syndrome, one of whom was found to have abnormal development of one side of the kidney during the prenatal examination, while the other had no additional special symptoms except the abnormality indicated by the imaging examination, was married and had children already. This article reviewed the patients’ medical history, main clinical manifestations, treatment process, various tests, pathological data and imaging findings. We also performed the analysis of the exon sequencing of these two patients to look for the genetic mutation associated with this disease.

## Case description

### Case 1

**Figure 1:**
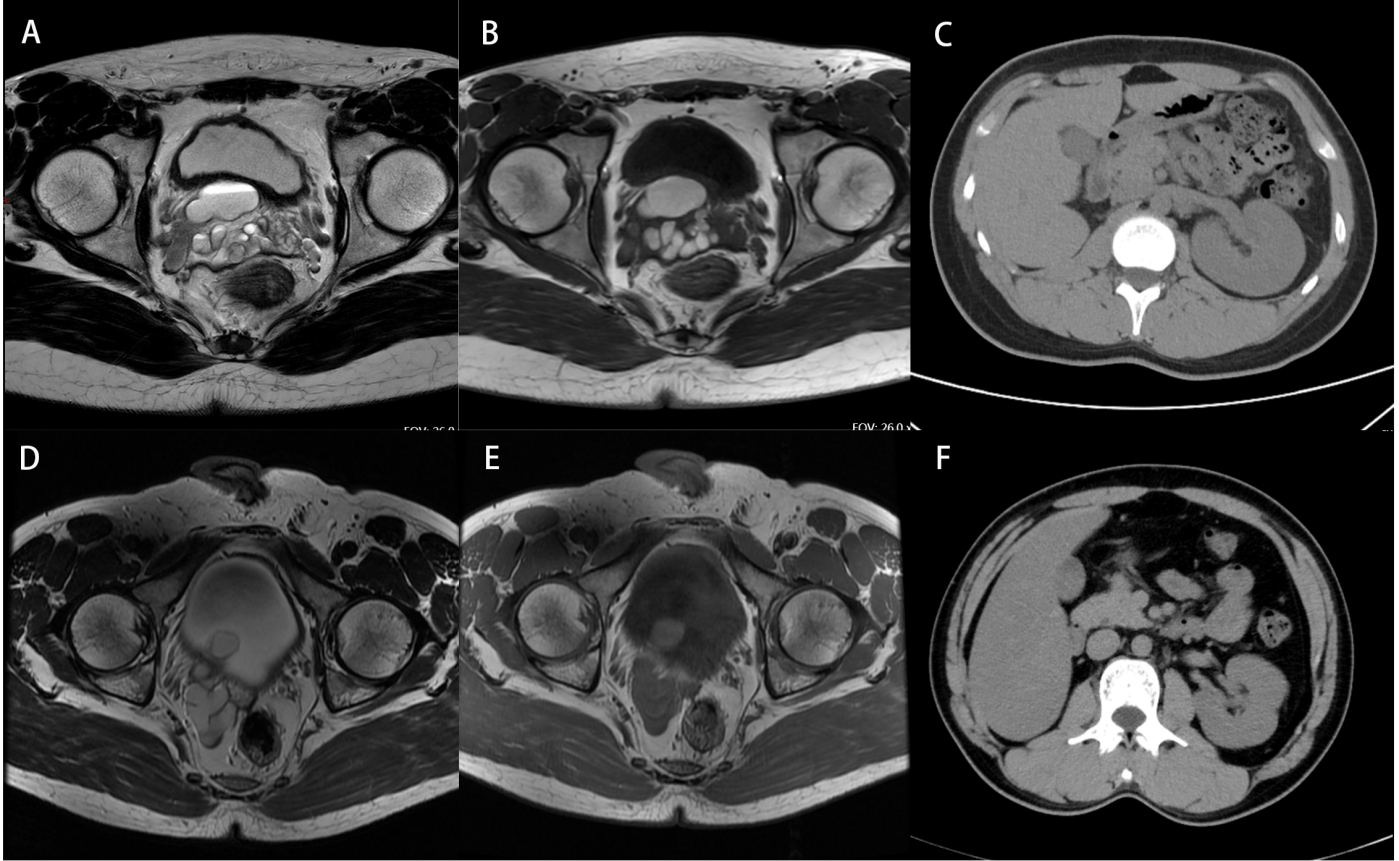
Pathological images of surgically removed glandular tissue of **(A–C)** case 1 and **(D–F)** case 2.

A 17-year-old male patient was admitted with a complaint of right renal dysplasia for 17 years and perineal pain for 1 week. No obvious lesions were found, as the patient’s external genitalia developed normally, and the bilateral scrotum was symmetrical. There was perineal distending pain accompanied by dysuria and painful urination, but the pain did not worsen when compressed. Transrectal ultrasonography showed that an oval cystic anechoic area of approximately 36*25 mm in size with a thin and smooth cyst wall could be detected at the base of the prostate, and the sonographer thought it could be an ejaculatory duct cyst. For further evaluation, the MRI scan results suggested that the right ureter was dilated with an end opening in the urethral prostatic part and that the right seminal vesicle gland was dilated with a seminal vesicle gland cyst. The same-day chest CT showed that the shadow of the right kidney was small and the left kidney was enlarged. The imaging data are shown in **Figure 1**. Taking a medical history revealed that the ultrasound examination of the patient’s mother during pregnancy indicated that the fetus was suspected of having a left solitary kidney. After that, the patient had a regular physical examination every year, except for dysplasia of the right kidney, and he had no other special uncomfortable symptoms. The patient first developed swelling and pain of the right testis with symptoms of the urinary system 2 years prior, and transrectal aspiration of pus was performed during the follow-up treatment. To further evaluate renal function, we performed renal dynamic imaging plus GFR measurement. The results showed that the right kidney could not be displayed, but the left renal blood perfusion and parenchyma function were approximately normal, GFR: L= 95.93 ml/min.

**Figure 2:**
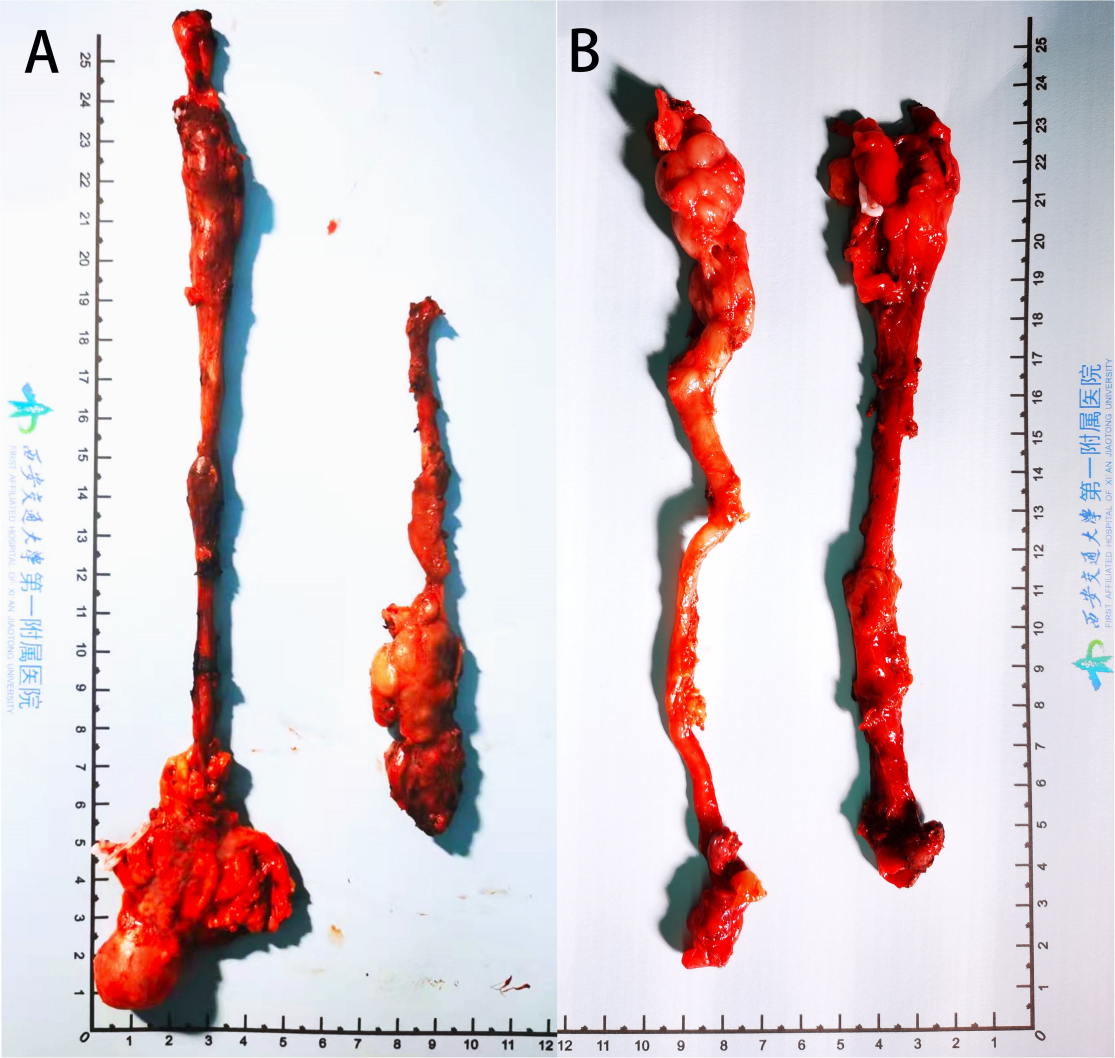
Surgically removed diseased tissue of **(A)** case 1 and **(B)** case 2.

**Figure 3:**
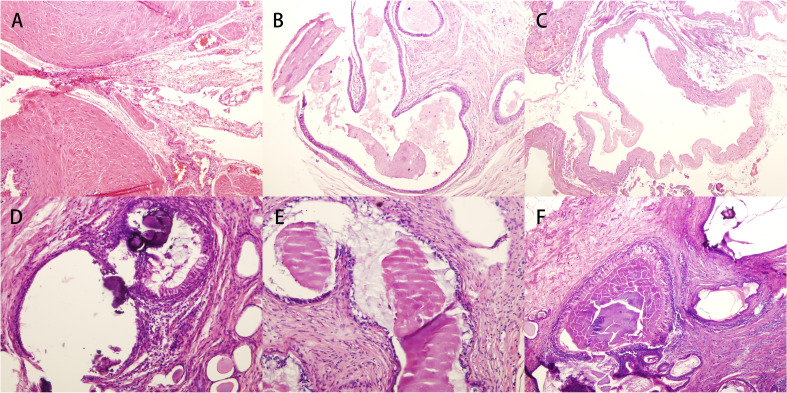
Imaging data of patients. **(A, B)** MRI of case 1 shows a right seminal vesicle cyst. **(C)** CT of case 1 shows a small right kidney and an enlarged left kidney outline. **(D, E)** MRI of case 2 shows a right seminal vesicle cyst combined with the right ectopic ureter and lower ureteral calculi. **(F)** CT of case 2 shows the absence of the right kidney.

After discussing the patient’s history, clinical manifestations and imaging findings, the diagnosis of Zinner’s syndrome was made in our hospital, and robot-assisted laparoscopic resection of the right kidney and ureter plus right seminal vesicle resection was selected as the operation method. The operation went smoothly with little intraoperative blood loss. The condition of the patient was stable, and he was discharged on the 3rd postoperative day. **Figure 2** shows the tissue resected during the operation, and the pathological images are shown in **Figure 3**. No postoperative complications have yet been reported.

### Case 2

A 32-year-old male patient was admitted to the hospital with a six-month complaint of a right seminal vesicle cyst found by physical examination. Physical examination after admission showed that the right testis was not palpated, while the external genitalia developed normally, and tenderness was not present in the perineum. The patient himself has never felt any discomfort symptoms to date. Following the medical history, we determined that the patient had a right orchiectomy for cryptorchidism 22 years prior. He was seen at the outpatient department of our hospital which revealed a right seminal vesicle cyst. Repeated urinary tract ultrasound showed atrophy of the right kidney whose location was in the pelvis and that the inner diameter of the right seminal vesicle was larger than that of the left seminal vesicle. To evaluate renal function, we performed renal dynamic imaging plus GFR measurement. The results showed that the left renal blood perfusion and parenchymal function were generally normal, GFR: L= 77.96 ml/min, while the right kidney could not be displayed, and a mass shadow with no radionuclide uptake distribution, which had a density equivalent to that of soft tissue, could be seen in the right seminal vesicle. Furthermore, the pelvic MRI scan showed that the right seminal vesicle cyst was combined with the right ectopic ureter and lower ureteral calculi, and only the left testis and spermatic cord were seen in the photo.

The diagnosis of Zinner’s syndrome was made based on the patient’s medical history, clinical manifestations and imaging findings. Then, we selected laparoscopic complete resection of the right kidney and ureter and resection of the right seminal vesicle cyst as the surgical procedure for this patient. The whole operation was successful, with approximately 50 ml of intraoperative blood loss in total. The patient recovered well and was discharged 6 days after the operation. No postoperative complications have been reported to date.

## Discussion

Zinner’s syndrome is a rare congenital disorder of the male urinary system, and the relevant literature suggests that the incidence rate of the population is only 0.0046% (3). In order to further evaluate the diagnosis and treatment of Zinner’s syndrome in China This paper summarizes 14 individual cases of Zinner’s syndrome reported in China in recent 10 years, as shown in **Table 1**.

**Table 1:** Reported cases of Zinner’s syndrome in the past few years.

Investigation	Years	Age	Main symptoms	Imaging examination before diagnosis	Imaging performance	Side	Size (mm)	Other genitourinary malformations	Coinfection	Treatment after diagnosis	Postoperative follow-up
Liao et al. (4)	2021	30	hematospermi, abdominal discomfort	US, CT, MRI	RKA, RSVC	right	62*50	no	no	TAUCA + sclerotherapy	NR
Cao et al. (5)	2017	23	perineal discomfort at the end of urination	CT, MRI	LKA, left cystic mass in prostate area	left	57*31	no	no	TRUCA	NR
Yu et al. (6)	2016	30	infertility	US, CT, MRI, cystoscope	LKA, left ureter mass near the bladder	left	30*32*33	no	no	LSVC	—
Lu et al. (7)	2019	24	abdominal discomfort and distention	CT	RKA, RSVC	right	70*30	right cryptorchidism	no	UCA + sclerotherapy	recurrence of SVC after 1 year
		36	pelvic mass revealed by medical examination	MRI	RKA, RSVC, right ureteral dilatation	right	100*80	no	no	LSVC + LUC	NR
Wang et al. (8)	2015	26	pollakisuria urgent urination, pain urination, constipate	US, CT, cystoscope IP	RKA, RSVC	right	42*35	no	no	LSVC + ureteral stump resection	NR
		31	perineal discomfort at the end of urination	US, CT, MRI	RKA, RSVC	right	83*53*40	no	right epididymitis	TUSVCI + SVWE	NR
Wang et al. (9)	2018	15	pollakisuria urgent urination, pain urination	US, MRI	LKA, LSVC, left ureteral cyst	left	120*50	no	no	RLSVC DER	NR
Ni et al. (10)	2022	18	pollakisuria pain urination, perineal pain	US, CT, MRI	LKA, LSVC	left	40*50	no	infection around bilateral seminal vesicles	LSVC	NR
Liao et al. (11)	2023	32	right cryptorchidism	US, CT	RKA, RSVC, right intra-abdominal cryptorchidism	right	32*24	right cryptorchidism	no	LC	—
Tan etc al. (12)	2021	19	perineal pain	CT, MRI, cystoscope	LKA, LSVC, left ureteral cyst	left	—	no	no	LSVC + LUC + HN	NR
Zhang et al. (13)	2016	52	pelvic mass revealed by medical examination	US, CT, MRI, IP	LKA, LSVC	left	68*36	no	no	LSVC	NR
Juho et al. (14)	2015	43	gastrointestinal upset, dry ejaculation, pollakisuria	CT, MRI, IP	RKD, RSVC	right	150*70	no	no	pelvic exploration + LSVC	NR
Liu et al. (15)	2020	23	pelvic mass revealed by medical examination	US, CT	LKA, left pelvic mass	left	80*80	no	no	robot-assisted Pelvic mass resection	NR

IP, intravenous pyelography; RKA, right kidney absence; RSVC, right seminal vesicle cyst; LKA, left kidney absence; LSVC, left seminal vesicle cyst; RKD, right kidney dysplasia; TAUCA, transabdominal ultrasound-guided cyst aspiration; TRUCA, transrectal ultrasound-guided cyst aspiration; LSVC, laparoscopic seminal vesicle cystectomy; UCA, ultrasound-guided cyst aspiration; TUSVCI, transurethral seminal vesicle cyst incision; SVWE, seminal vesicle wall electrocautery; RLSVC, robot-assisted laparoscopic seminal vesicle cystectomy; DER, deferens and ejaculatory duct reconstruction; LC, laparoscopic cryptorchidectomy; LUC, laparoscopic ureteral cystectomy; HN, hypoplastic nephrectomy; NR, no recurrence.

It is currently believed that the disease is caused by abnormal growth of the distal Wolffian duct during the 4th and 13th weeks of gestation. This abnormal development results in the failure of the ureteral bud to migrate and combine with the metanephros to form the kidney, and the end of the mesonephric duct is also deformed (16). During the 3rd and 8th week of gestation, paired mesonephric ducts and paramesonephric ducts are present in both male and female embryos. The ureteral buds arise from the dorsal aspect of the distal mesonephric ducts at the 4th week of gestation and migrate in a dorsocranial fashion to meet and induce differentiation of the metanephric blastema, which will form the definitive adult kidney. In males, under the influence of testosterone and Mullerian inhibitory factor, the paramesonephric ducts regress and the caudal part of mesonephric ducts differentiates into hemitrigone, bladder neck, urethra up to the external sphincter, seminal vesicle, vas deferens, ejaculatory ducts, and epididymis. Maldevelopment of the distal mesonephric duct results in absence or malposition of the ureteral budding, and therefore, ipsilateral renal agenesis or dysplasia. Also, it may lead to malformation of mesonephric duct derivatives (17, 18). However, no studies have focused on the genetic changes in patients with Zinner’s syndrome. As a congenital developmental deformity, is there some certain genes closely related to the onset of the disease? In order to explore this problem, we conducted whole exome sequencing on these two patients to obtain the tested results of single nucleotide variants (SNVs) and insertions or deletions (InDels) that met the quality control requirements. Software Annovar (19) was used to annotate the results in terms of location, frequency, gene function, toxicity and so on. Then filter out the mutations which the frequency of the 1000 Genomes Project database > 0.01 and the ExAC (Exome Aggregation Consortium) database in east Asia > 0.01 in the crowd, eliminate the individual diversity and remain possibly pathogenic rare mutations. After that, variation in the exon region or splicing site region is retained, and the synonym mutation is removed to obtain which have an impact on gene expression products. Finally, the mutation that be predicted had an impact on protein structure or function by more than two tools in CADD (20), SIFT (21), Polyphen2 (22) and MutationTaster2 (23) were selected. After the above steps, the potentially meaningful mutation sites were finally screened respectively. Considering that Zinner’s syndrome is a congenital developmental disorder, germ line/developmental related mutation were further selected from the above screened and presented in the **Supplementary Tables 1–4**. The germ line/developmental related mutations detected in both cases are listed in **Table 2**. It is worth noting that the BMP4, RPGRIP1L SEC63, SETD2 as genes associated with urinary system morphogenesis or development, are mutated in both cases.

**Table 2:** The germ-line/developmental-related gene mutations detected in both cases.

Gene name	Chromosome region	Region of mutation	Predicted effect to the mutation
NES	1q23.1	exonic	central nervous system/embryonic camera-type eye development
SEC63	6q21	splicing	liver/renal system development
RPGRIP1L	16q12.2	splicing	in utero embryonic/kidney/liver/pericardium/nervous system development; embryonic forelimb/hindlimb morphogenesis
FLT1	13q12.3	exonic	angiogenesis; embryonic morphogenesis
ZNF141	4p16.3	exonic	anatomical structure/limb morphogenesis
FLG	1q21.3	exonic	multicellular organism development; skin epidermis development
BMP4	14q22.2	exonic	endoderm/ureteric bud/kidney/mesonephros/cardiovascular/lung/hematopoietic system development; neural tube closure
SETD2	3p21.31	exonic	angiogenesis; mesoderm/embryonic placenta/branching structure morphogenesis; neural tube closure; pericardium development
AGFG1	2q36.3	splicing	multicellular organism development

The syndrome is mainly clinically manifested as seminal vesicle cyst, ejaculatory duct obstruction, and ipsilateral renal agenesia (24, 25). The corresponding symptoms of Zinner’s syndrome are atypical, including ejaculation disturbance, local pain, bladder irritation, epididymitis or prostatitis and infertility (1, 26). According to cases reported in China in recent 10 years, discomfort during urination, such as pollakisuria, urgent and painful urination, is the most common symptom, followed by pain in the perineum or lower abdomen, and other non-specific urinary system symptoms such as infertility, dry ejaculation, blood spermia and digestive system discomfort also account for a certain proportion. In addition to patients with corresponding clinical symptoms, there are nearly 1/5 patients without any discomforts. Of the two patients reported in this paper, the younger one had obvious symptoms of perineal and lower abdominal pain and urination discomfort, while the other did not have any noticeable discomfort symptoms, but only reported abnormalities in the results of physical examination.

On imaging, almost every patient with Zinner’s syndrome will have unilateral renal absence and seminal vesicle cyst, which is also the most important and diagnostic imaging manifestations of Zinner’s syndrome. Nearly half of patients have abnormal ureteral dilatation that may be manifested as an X-ray mass in the pelvic region. Generally, in the imaging examination, patients with unilateral absence of kidney and abnormal mass in the ipsilateral pelvis should consider the diagnosis of Zinner’s syndrome.

From the summary and analysis results, the clinical symptoms and imaging findings of Zinner’s syndrome are atypical, which is one of the reasons for misdiagnosis. Therefore, for patients with above atypical urinary symptoms and corresponding imaging findings, attention should be paid to the diagnosis of Zinner’s syndrome. However, there were patients with no specific symptoms other than imaging abnormalities in case 2 and three patients in **Table 1**. For this kind of patients, imaging examination is the most important diagnostic method. A small number of patients have cryptorchidism in addition to the above clinical symptoms and imaging findings, such as case 2. Is cryptorchidism also related to the congenital dysplasia of the disease? The diagnosis of Zinner’s syndrome is mainly based on the results of clinical symptoms and imaging examinations. Common examinations include transrectal or transabdominal ultrasonography, CT scan, and magnetic resonance imaging (27, 28); sometimes cystoscopy can also find abnormal manifestations. A cystic change of approximately 2*2 cm in size was seen in the right ureteral orifice through cystoscopy during bladder examination of the second patient in this paper. Therefore, care should be taken to avoid misdiagnosis when cystoscopy suggests abnormalities in patients with urologic symptoms.

In terms of disease treatment for patients with obvious symptoms of discomfort, the main principle of Zinner’s syndrome is still symptomatic treatment to reduce the symptoms caused by the mass effect of seminal vesicle cysts (24), including more conservative treatments such as cyst aspiration and sclerotherapy, as well as radical resection (28, 29). However, the results of the reported cases in recent 10 years show that cyst aspiration and sclerotherapy have a greater chance of recurrence than excisional surgery. Cyst aspiration and sclerotherapy has the advantages of convenient operation and less trauma, but the treatment is not thorough and the recurrence rate remains higher; although the recurrence rate after open radical resection is low, because of the deep position of seminal vesicle gland, there is a higher risk of injury to bladder, rectum and other organs during the operation, and the postoperative recovery time will also be prolonged. With the development of minimally invasive techniques, especially robot-assisted surgery, in recent years, the high risk of structural damage to the bladder neck, external sphincter, and rectum that may be caused by open surgery has been significantly reduced (30). In case 1 of this article, the parents of the patient considered that he was young and had not yet married or had children, so they chose robot-assisted laparoscopic surgery with more detailed dissection of the deep pelvic location as well as less surgical trauma.

## Conclusion

As a rare disease with a low incidence, Zinner’s syndrome has very different clinicopathological manifestations, and in most cases, the symptoms are nonspecific. These factors lead to the clinical misdiagnosis and neglect of the disease. Therefore, in the process of diagnosis and treatment, patients with specific imaging manifestations should be aware that their disease may be Zinner’s syndrome to avoid misdiagnosis and mistreatment. Although this study filled the gap in the genetic research of Zinner’s syndrome to a certain extent, the number of included cases was too small to obtain any statistical significance. The sample number of cases needed to be further expanded.

## Data availability statement

The original contributions presented in the study are included in the article/**Supplementary Material**. Further inquiries can be directed to the corresponding author.

## Ethics statement

Written informed consent was obtained from the individual(s) for the publication of any potentially identifiable images or data included in this article.

## Author contributions

RD: Writing – original draft. KW: Writing – review & editing. FJ: Writing – review & editing. JF: Writing – review & editing. DH: Writing – review & editing. LL: Writing – review & editing.
