# Supplementary material for: Clinicopathological and genetic features of Zinner’s syndrome: two case reports and review of the literature
Source: Front Urol. 2023 Oct 19;3:1257368. doi: 10.3389/fruro.2023.1257368 (PMC12327280; doi:10.3389/fruro.2023.1257368)
Supplement: Supplementary file 1 [file Table_1.docx]

**Supplementary Table 1.** The germ-line/developmental-related InDels detected in case 1.

| Gene name | Chromosome region | Region of mutation | Types of exon region mutations | Amino acid changes | Predicted effect to the mutation |
| --- | --- | --- | --- | --- | --- |
| FLG | 1q21.3 | exonic | frameshift deletion | p.A2865fs | multicellular organism development; skin epidermis development |
| FLG | 1q21.3 | exonic | frameshift deletion | p.H2864fs | multicellular organism development; skin epidermis development |
| FLG | 1q21.3 | exonic | frameshift deletion | p.A920fs | multicellular organism development; skin epidermis development |
| FLG | 1q21.3 | exonic | frameshift deletion | p.H919fs | multicellular organism development; skin epidermis development |
| NES | 1q23.1 | exonic | nonframeshift deletion | p.1100_1101del | central nervous system development |
| AGFG1 | 2q36.3 | splicing | — | — | multicellular organism development |
| ZNF141 | 4p16.3 | exonic | frameshift deletion | p.H68fs | anatomical structure/limb morphogenesis |
| ZNF141 | 4p16.3 | exonic | frameshift deletion | p.I70fs | anatomical structure/limb morphogenesis |
| TRIML1 | 4q35.2 | exonic | frameshift deletion | p.S361fs | multicellular organism development |
| PRDM6 | 5q23.2 | exonic | nonframeshift deletion | p.51_52del | Neurogenesis |
| SEC63 | 6q21 | splicing | — | — | liver/renal system development |
| SEC63 | 6q21 | splicing | — | — | liver/renal system development |
| SPATA31C2 | 9q22.1 | exonic | nonframeshift deletion | p.P71delinsHLVSQRHLVSQRP | spermatogenesis; cell differentiation |
| C5 | 9q33.2 | splicing | — | — | in utero embryonic development |
| RPGRIP1L | 16q12.2 | splicing | — | — | in utero embryonic/kidney/liver/nervous system development |
| VCX | Xp22.31 | exonic | frameshift deletion | p.Q129fs | spermatogenesis; brain development |
| VCX | Xp22.31 | exonic | frameshift deletion | p.V130fs | spermatogenesis; brain development |

**Supplementary Table 2.** The germ-line/developmental-related SNVs detected in case 1.

| Gene name | Chromosome region | Region of mutation | Types of exon region mutations | Amino acid changes | Predicted effect to the mutation |
| --- | --- | --- | --- | --- | --- |
| MFN2 | 1p36.22 | exonic | nonsynonymous SNV | p.F661V | blastocyst formation; camera-type eye morphogenesis |
| WLS | 1p31.3 | splicing | — | — | mesoderm formation; brain/exocrine pancreas development |
| RBM15 | 1p13.3 | exonic | nonsynonymous SNV | p.I247V | spleen/ventricular septum/placenta blood vessel development |
| FLG | 1q21.3 | exonic | nonsynonymous SNV | p.N3534S | multicellular organism development; skin epidermis development |
| FLG | 1q21.3 | exonic | nonsynonymous SNV | p.T3531R | multicellular organism development; skin epidermis development |
| FLG | 1q21.3 | exonic | nonsynonymous SNV | p.S3160R | multicellular organism development; skin epidermis development |
| FLG | 1q21.3 | exonic | nonsynonymous SNV | p.T2863S | multicellular organism development; skin epidermis development |
| FLG | 1q21.3 | exonic | nonsynonymous SNV | p.G929V | multicellular organism development; skin epidermis development |
| NES | 1q23.1 | exonic | nonsynonymous SNV | p.L1108R | central nervous system development |
| NES | 1q23.1 | exonic | nonsynonymous SNV | p.V1105G | central nervous system development |
| NES | 1q23.1 | exonic | nonsynonymous SNV | p.Q1103R | central nervous system development |
| NES | 1q23.1 | exonic | nonsynonymous SNV | p.Q1103E | central nervous system development |
| CENPF | 1q41 | exonic | nonsynonymous SNV | p.K1845N | kidney/muscle organ/ventricular system development |
| HLX | 1q41 | exonic | nonsynonymous SNV | p.Q222L | multicellular organism development; liver/skeletal muscle tissue/digestive tract development |
| PKDCC | 2p21 | exonic | nonsynonymous SNV | p.R300H | multicellular organism growth; embryonic digestive tract/skeletal system/lung development; limb morphogenesis |
| MYO7B | 2q14.3 | exonic | nonsynonymous SNV | p.R206C | cell differentiation |
| AMER3 | 2q21.1 | exonic | nonsynonymous SNV | p.R130H | anatomical structure development |
| LRP2 | 2q31.1 | exonic | nonsynonymous SNV | p.R3305H | neural tube closure; cardiovascular system/gonad/forebrain/vagina development |
| HOXD4 | 2q31.1 | exonic | nonsynonymous SNV | p.G68R | multicellular organism development; embryonic skeletal system morphogenesis |
| SETD2 | 3p21.31 | exonic | nonsynonymous SNV | p.R1278Q;p.R1322Q | endodermal cell differentiation; angiogenesis; branching structure/embryonic placenta/mesoderm morphogenesis; neural tube closure |
| HEG1 | 3q21.2 | exonic | nonsynonymous SNV | p.T811I | vasculogenesis; endothelial cell morphogenesis; in utero embryonic/lymph vessel/cardiovascular system/lung development |
| ADGRV1 | 5q14.3 | exonic | nonsynonymous SNV | p.R5246Q | nervous system development |
| COL19A1 | 6q13 | exonic | nonsynonymous SNV | p.G1115D | skeletal system development; cell differentiation |
| SEMA3C | 7q21.11 | splicing | — | — | post-embryonic/neural tube/cardiovascular system development; limb bud formation |
| NRG1 | 8p12 | splicing | — | — | cell morphogenesis; embryo/cardiovascular system/nervous system development |
| ADAM18 | 8p11.22 | exonic | nonsynonymous SNV | p.M563R;p.M587R | multicellular organism development; spermatogenesis; cell differentiation |
| ROR2 | 9q22.31 | exonic | nonsynonymous SNV | p.R638Q | multicellular organism/genitalia development; embryonic genitalia/inner ear/digit morphogenesis |
| COL5A1 | 9q34.3 | exonic | nonsynonymous SNV | p.P656L | heart morphogenesis; blood vessel/tendon/skin development; endodermal cell differentiation |
| RET | 10q11.21 | exonic | nonsynonymous SNV | p.P425L;p.P679L | ureteric bud/nervous system/renal system/camera-type eye development |
| DCHS1 | 11p15.4 | exonic | nonsynonymous SNV | p.R2964C | neurogenesis; ureteric bud morphogenesis; cardiovascular system/neural tube/digestive tract/cochlea development |
| ARID2 | 12q12 | exonic | nonsynonymous SNV | p.I723V | heart morphogenesis; embryonic organ development |
| COL2A1 | 12q13.11 | splicing | — | — | heart morphogenesis; skeletal system/central nervous system/palate development; limb bud formation |
| GLI1 | 12q13.3 | exonic | nonsynonymous SNV | p.G500R;p.G587R;p.G628R | ventral midline/nervous system/lung/prostate gland development |
| MMP17 | 12q24.33 | exonic | nonsynonymous SNV | p.G214S | kidney development |
| FLT1 | 13q12.3 | exonic | nonsynonymous SNV | p.R593Q | angiogenesis; cell differentiation; embryonic morphogenesis |
| BMP4 | 14q22.2 | exonic | nonsynonymous SNV | p.R162Q;p.R99Q;p.R209Q | endoderm/ureteric bud/kidney/mesonephros/cardiovascular/lung/hematopoietic system development; neural tube closure |
| BTBD7 | 14q32.12 | exonic | nonsynonymous SNV | p.E226Q | multicellular organism development |
| TDRD9 | 14q32.33 | exonic | nonsynonymous SNV | p.S533G | multicellular organism development; spermatogenesis |
| SPINT1 | 15q15.1 | exonic | nonsynonymous SNV | p.R438Q;p.R454Q | neural tube closure; placenta blood vessel/nervous system development |
| RBBP6 | 16p12.1 | exonic | nonsynonymous SNV | p.R1463Q;p.R1497Q | in utero embryonic/embryonic organ/somite development |
| ACE | 17q23.3 | exonic | nonsynonymous SNV | p.T313N;p.T887N | kidney development; spermatogenesis |
| NLRP5 | 19q13.43 | exonic | nonsynonymous SNV | p.T1073M | in utero embryonic development; animal organ morphogenesis |

**Supplementary Table 3.** The germ-line/developmental-related InDels detected in case 2.

| Gene name | Chromosome region | Region of mutation | Types of exon region mutations | Amino acid changes | Predicted effect to the mutation |
| --- | --- | --- | --- | --- | --- |
| NES | 1q23.1 | exonic | frameshift deletion | p.L1108fs | central nervous system/embryonic camera-type eye development |
| NES | 1q23.1 | exonic | frameshift deletion | p.Q1103fs | central nervous system/embryonic camera-type eye development |
| NES | 1q23.1 | exonic | frameshift deletion | p.Q1103fs | central nervous system/embryonic camera-type eye development |
| MSGN1 | 2p24.2 | exonic | frameshift deletion | p.G77fs | mesoderm formation; embryonic pattern specification |
| AGFG1 | 2q36.3 | splicing | — | — | multicellular organism development |
| SEC63 | 6q21 | splicing | — | — | liver/renal system development |
| SEC63 | 6q21 | splicing | — | — | liver/renal system development |
| MPZL2 | 11q23.3 | exonic | nonframeshift deletion | p.25_26del | anatomical structure morphogenesis |
| RPGRIP1L | 16q12.2 | splicing | — | — | in utero embryonic/kidney/liver/pericardium/nervous system development; embryonic forelimb/hindlimb morphogenesis |
| ZMYM3 | Xq13.1 | exonic | nonframeshift deletion | p.P804delinsAPPPP;p.P816delinsAPPPP | multicellular organism development; regulation of cell morphogenesis |

**Supplementary Table 4.** The germ-line/developmental-related SNVs detected in case 2.

| Gene name | Chromosome region | Region of mutation | Types of exon region mutations | Amino acid changes | Predicted effect to the mutation |
| --- | --- | --- | --- | --- | --- |
| ECE1 | 1p36.12 | exonic | nonsynonymous SNV | p.V642M;p.V651M;p.V638M;p.V654M | ear/heart/pharyngeal system development; embryonic digit morphogenesis |
| HSPG2 | 1p36.12 | exonic | nonsynonymous SNV | p.E147A | angiogenesis; brain/cardiovascular system development |
| TGFBR3 | 1p22.1 | splicing | — | — | liver/cardiovascular system development |
| FLG | 1q21.3 | exonic | nonsynonymous SNV | p.N3534S | multicellular organism development; skin epidermis development |
| FLG | 1q21.3 | exonic | nonsynonymous SNV | p.T3531R | multicellular organism development; skin epidermis development |
| BCAN | 1q23.1 | exonic | nonsynonymous SNV | p.R851S | skeletal system/central nervous system development |
| PRELP | 1q32.1 | exonic | nonsynonymous SNV | p.R95Q | skeletal system development; axonogenesis |
| OBSCN | 1q42.13 | exonic | nonsynonymous SNV | p.R665W | multicellular organism development |
| OBSCN | 1q42.13 | exonic | nonsynonymous SNV | p.T4223M;p.T5180M | multicellular organism development |
| OBSCN | 1q42.13 | exonic | nonsynonymous SNV | p.L4820P;p.L5777P | multicellular organism development |
| OBSCN | 1q42.13 | exonic | nonsynonymous SNV | p.R5276H;p.R5276H;p.R6233H | multicellular organism development |
| MTR | 1q43 | splicing | — | — | nervous system development |
| AFF3 | 2q11.2 | exonic | nonsynonymous SNV | p.A160T;p.A135T | embryonic hindlimb morphogenesis |
| NEB | 2q23.3 | exonic | nonsynonymous SNV | p.A4462V;p.A6163V | muscle organ development |
| ITGA6 | 2q31.1 | exonic | nonsynonymous SNV | p.R942W;p.R823W;p.R927W | nail/skin/renal system development |
| TTN | 2q31.2 | exonic | nonsynonymous SNV | p.A4564T;p.A5491T;p.A5808T | cardiac muscle tissue morphogenesis |
| TTN | 2q31.2 | exonic | nonsynonymous SNV | p.S4273L;p.S5200L;p.S5517L | cardiac muscle tissue morphogenesis |
| ALS2 | 2q33.1 | exonic | nonsynonymous SNV | p.E159K | in utero embryonic development; axonogenesis |
| SETD2 | 3p21.31 | exonic | nonsynonymous SNV | p.H172D;p.H216D | angiogenesis; mesoderm/embryonic placenta/branching structure morphogenesis; neural tube closure; pericardium development |
| FLNB | 3p14.3 | exonic | nonsynonymous SNV | p.V1195M | epithelial cell morphogenesis |
| ZNF141 | 4p16.3 | exonic | nonsynonymous SNV | p.S276T;p.S352T | anatomical structure/limb morphogenesis |
| ENAM | 4q13.3 | exonic | nonsynonymous SNV | p.D807E;p.D1025E | regulation of cell morphogenesis |
| FRAS1 | 4q21.21 | exonic | nonsynonymous SNV | p.S2190F | epithelium/metanephros/embryonic limb morphogenesis; skin/palate development |
| ADAD1 | 4q27 | exonic | nonsynonymous SNV | p.M315V;p.M308V;p.M326V | multicellular organism development |
| DNAH5 | 5p15.2 | exonic | nonsynonymous SNV | p.R1245C | heart/lateral ventricle development |
| PRDM1 | 6q21 | exonic | nonsynonymous SNV | p.P333L;p.P467L | morphogenesis of a branching structure; cardiovascular system/germ cell/post-embryonic development |
| AHI1 | 6q23.3 | splicing | — | — | hindbrain/cloaca/renal system/otic vesicle development |
| CHD7 | 8q12.2 | exonic | nonsynonymous SNV | p.T730I | skeletal system/in utero embryonic/cardiovascular system/central nervous system/genitalia development |
| ZFPM2 | 8q23.1 | exonic | nonsynonymous SNV | p.T792M;p.T713M:p.T845M | vasculogenesis; in utero embryonic/cardiovascular system/gonadal mesoderm/lung development |
| HSF1 | 8q24.3 | exonic | nonsynonymous SNV | p.G149E | embryonic placenta development; positive regulation of multicellular organism growth |
| HSF1 | 8q24.3 | exonic | nonsynonymous SNV | p.L473M | embryonic placenta development; positive regulation of multicellular organism growth |
| FOXD4L3 | 9q21.11 | exonic | nonsynonymous SNV | p.S84L | anatomical structure morphogenesis; cell differentiation |
| AK8 | 9q34.13 | exonic | nonsynonymous SNV | p.L155F;p.L359F;p.L187F | ventricular system development |
| LHX3 | 9q34.3 | splicing | — | — | placenta/pituitary gland/lung development; animal organ morphogenesis |
| CREM | 10p11.21 | exonic | nonsynonymous SNV | p.T85N;p.T98N;p.T102N;p.T127N;p.T114N;p.T110N;p.T105N;p.T171N;p.T196N;p.T234N;p.T259N;p.T210N;p.T168N;p.T271N;p.T289N | multicellular organism development |
| ERCC6;PGBD3 | 10q11.23 | exonic | nonsynonymous SNV | p.T93M;p.T561M | multicellular organism growth |
| PCDH15 | 10q21.1 | exonic | nonsynonymous SNV | p.H1845N;p.H1816N;p.H1882N;p.H1862N;p.H1865N;p.H1885N;p.H1887N;p.H1892N;p.H1863N | nervous system/inner ear development |
| SEMA4G | 10q24.31 | exonic | nonsynonymous SNV | p.R415S | nervous system development |
| PDGFD | 11q22.3 | exonic | nonsynonymous SNV | p.S271R;p.S265R | multicellular organism development |
| CACNA1C | 12p13.33 | splicing | — | — | immune system/heart development; embryonic forelimb morphogenesis |
| CACNA1C | 12p13.33 | exonic | nonsynonymous SNV | p.T1878M;p.T1876M;p.T1859M;p.T1870M;p.T1911M;p.T1889M;p.T1887M;p.T1867M;p.T1930M;p.T1905M;p.T1898M;p.T1890M;p.T1918M;p.T1953M | immune system/heart development; embryonic forelimb morphogenesis |
| ATP8A2 | 13q12.13 | exonic | nonsynonymous SNV | p.A1004T;p.A1069T | axonogenesis |
| FLT1 | 13q12.3 | exonic | nonsynonymous SNV | p.R312C | angiogenesis; embryonic morphogenesis |
| BMP4 | 14q22.2 | exonic | nonsynonymous SNV | p.R162Q;p.R99Q;p.R162Q;p.R209Q | endoderm/ureteric bud/kidney/mesonephros/cardiovascular/lung/hematopoietic system development; neural tube closure |
| PGF | 14q24.3 | exonic | nonsynonymous SNV | p.P24R | angiogenesis; branching involved in ureteric bud morphogenesis |
| DUOX2 | 15q21.1 | exonic | nonsynonymous SNV | p.R885Q | multicellular organism growth; cuticle/thyroid gland development; |
| PKD1 | 16p13.3 | exonic | nonsynonymous SNV | p.A3980V;p.A3981V | in utero embryonic/embryonic placenta/kidney/liver/heart/spinal cord/neural tube development; anatomical structure morphogenesis |
| UNC45B | 17q12 | exonic | nonsynonymous SNV | p.Q131H | lens development in camera-type eye; muscle organ development |
| KLHL26 | 19p13.11 | exonic | nonsynonymous SNV | p.I590M;p.I471M;p.I561M;p.I550M | spermatid/male gonad development |
| WDR62 | 19q13.12 | exonic | nonsynonymous SNV | p.R1230C;p.R1225C | neurogenesis |
| GDF5 | 20q11.22 | exonic | nonsynonymous SNV | p.R318P | embryonic limb morphogenesis; multicellular organism growth; |
| MECP2 | Xq28 | exonic | nonsynonymous SNV | p.T323M;p.T218M;p.T311M | post-embryonic development; cerebellum/ventricular system development; cardiolipin metabolic process |
